# 4-Chloro-*N*-(3,4,5-trimethoxy­benzyl­idene)aniline

**DOI:** 10.1107/S1600536809000300

**Published:** 2009-01-08

**Authors:** Aliakbar Dehno Khalaji, Jilla Asghari, Karla Fejfarová, Michal Dušek

**Affiliations:** a Department of Science, Gorgan University of Agricultural Sciences and Natural Resources, Gorgan 49189-43464, Iran; b Institute of Physics of the ASCR, Na Slovance 2, 182 21 Prague 8, Czech Republic

## Abstract

The title compound, C_16_H_16_ClNO_3_, is a Schiff base displaying a *trans* configuration of the C=N double bond. In the crystal structure, inter­molecular C—H⋯N and bifurcated C—H⋯(O,O) hydrogen bonds are observed.

## Related literature

For backgroud and related structures, see: Khalaji *et al.* (2008 ▶); Khalaji & Harrison (2008 ▶); Khalaji *et al.* (2007 ▶); Zhang (2008 ▶); Akkurt *et al.* (2008 ▶); Kashmiri *et al.* (2008 ▶); Ren & Jian (2008 ▶). For the synthesis of the title compound, see: Khalaji & Ng (2008 ▶).
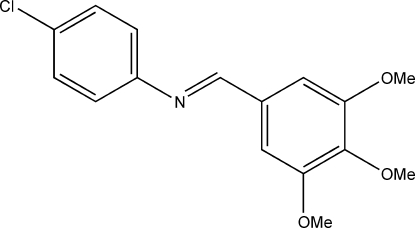


## Experimental

### 

#### Crystal data

C_16_H_16_ClNO_3_
*M* *_r_* = 305.75
Monoclinic, 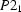

*a* = 7.2012 (2) Å
*b* = 8.18700 (10) Å
*c* = 12.9734 (3) Å
β = 105.050 (2)°
*V* = 738.63 (3) Å^3^
*Z* = 2
Cu *K*α radiation
μ = 2.37 mm^−1^
*T* = 120 K
0.22 × 0.20 × 0.11 mm

#### Data collection

Oxford Diffraction Gemini diffractometer
Absorption correction: numerical [Clark & Reid (1995 ▶) in *CrysAlis RED* (Oxford Diffraction, 2008 ▶)] *T*_min_ = 0.680, *T*_max_ = 0.809
5670 measured reflections
2225 independent reflections
2039 reflections with *I* > 3σ(*I*)
*R*_int_ = 0.048

#### Refinement

*R*[*F* ^2^ > 2σ(*F* ^2^)] = 0.045
*wR*(*F* ^2^) = 0.109
*S* = 1.93
2225 reflections
189 parameters
H-atom parameters constrained
Δρ_max_ = 0.28 e Å^−3^
Δρ_min_ = −0.21 e Å^−3^
Absolute structure: Flack (1983 ▶), 915 Friedel pairs
Flack parameter: 0.06 (2)

### 

Data collection: *CrysAlis CCD* (Oxford Diffraction, 2005 ▶); cell refinement: *CrysAlis RED* (Oxford Diffraction, 2008 ▶); data reduction: *CrysAlis RED*; program(s) used to solve structure: *SIR2002* (Burla *et al.*, 2003 ▶); program(s) used to refine structure: *JANA2006* (Petříček *et al.*, 2007 ▶); molecular graphics: *DIAMOND* (Brandenburg & Putz, 2005 ▶); software used to prepare material for publication: *JANA2006*.

## Supplementary Material

Crystal structure: contains datablocks global, I. DOI: 10.1107/S1600536809000300/bt2844sup1.cif

Structure factors: contains datablocks I. DOI: 10.1107/S1600536809000300/bt2844Isup2.hkl

Additional supplementary materials:  crystallographic information; 3D view; checkCIF report

## Figures and Tables

**Table 1:** Hydrogen-bond geometry (Å, °)

*D*—H⋯*A*	*D*—H	H⋯*A*	*D*⋯*A*	*D*—H⋯*A*
C7—H7⋯O1^i^	0.96	2.59	3.177 (4)	119
C7—H7⋯O2^i^	0.96	2.51	3.471 (4)	178
C12—H12⋯N1^ii^	0.96	2.61	3.545 (5)	164

Symmetry codes: (i) 
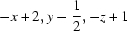
; (ii) 
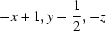
.

**Fig. 1.**
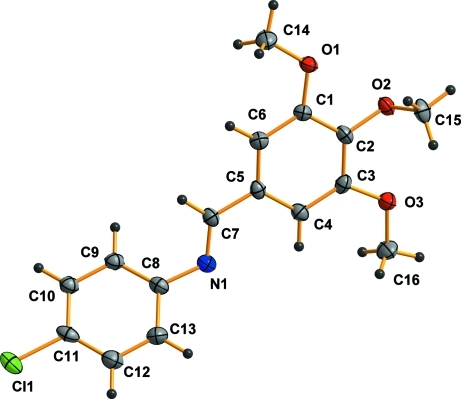
The molecular structure of the title compound with atom-labeling scheme. Displacement ellipsoids are drawn at the 50% probability level.

**Fig. 2.**
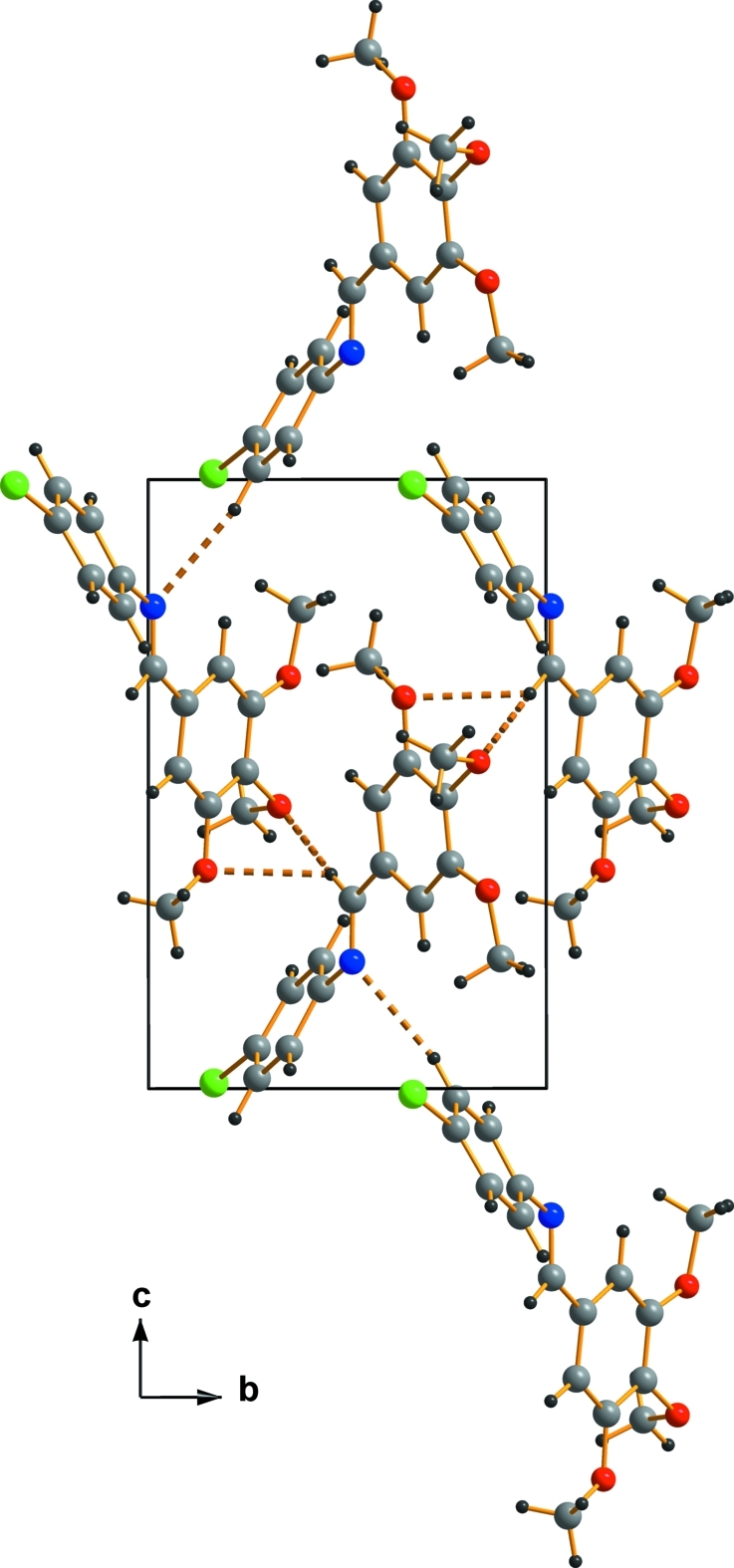
The packing of (I) viewed along a, with hydrogen bonds shown as dashed lines.

## supplementary crystallographic information

### Comment

Studies on the Schiff-base compounds, products of condensation between aldehydes (or ketones) and amines, have received a lot of attention in recent years, (Khalaji *et al.*, 2008; Khalaji & Harrison, 2008; Khalaji *et al.*,2007; Zhang, 2008; Akkurt *et al.*, 2008; Kashmiri *et al.*, 2008; Ren & Jian, 2008). As a continuation of these studies we present the crystal structure of C_16_H_16_ClNO_3_.

The molecular structure of the title compound is shown in Fig. 1. Bond lengths and angles are comparable with those observed in similar compounds (Khalaji *et al.*, 2008; Khalaji & Harrison, 2008; Khalaji *et al.*,2007; Zhang, 2008; Akkurt *et al.*, 2008; Kashmiri *et al.*, 2008; Ren & Jian, 2008). In the crystal structure, intermolecular C—H···N and C—H···O hydrogen bonds are observed.

### Experimental

The title compound  was synthesized using a method analogous to the literature procedure of Khalaji and Ng (2008). Crystals appropriate for data collection were obtained by slow evaporation from methanol-chloroform (1:5 *v/v*) at a room temperature (yield 83%).

### Refinement

All the H atoms were found in difference Fourier maps, but they were constrained to ideal positions. The isotropic atomic displacement parameters of hydrogen atoms were set to 1.2*U*_eq_ of the parent atom.

### Crystal data

**Table:**

C_16_H_16_ClNO_3_	*F*(000) = 320
*M**_r_* = 305.75	*D*_x_ = 1.379 Mg m^−^^3^
Monoclinic, *P*2_1_	Cu *K*α radiation, λ = 1.54184 Å
Hall symbol: P 2yb	Cell parameters from 4239 reflections
*a* = 7.2012 (2) Å	θ = 3.5–62.6°
*b* = 8.1870 (1) Å	µ = 2.37 mm^−^^1^
*c* = 12.9734 (3) Å	*T* = 120 K
β = 105.050 (2)°	Prism, colorless
*V* = 738.63 (3) Å^3^	0.22 × 0.20 × 0.11 mm
*Z* = 2	

### Data collection

**Table:**

Oxford Diffraction Gemini diffractometer with Xcalibur goniometer, Atlas detector and Gemini ultra Cu source	2225 independent reflections
Radiation source: X-ray tube	2039 reflections with *I* > 3σ(*I*)
mirror	*R*_int_ = 0.048
Detector resolution: 20.7567 pixels mm^-1^	θ_max_ = 62.7°, θ_min_ = 3.5°
rotation method data acquisition using ω scans	*h* = −8→7
Absorption correction: numerical [based on the crystal shape, using the method implemented in *CrysAlis RED* (Oxford Diffraction, 2008) according to Clark & Reid (1995)]	*k* = −9→9
*T*_min_ = 0.680, *T*_max_ = 0.809	*l* = −14→14
5670 measured reflections	

### Refinement

**Table:**

Refinement on *F*^2^	H-atom parameters constrained
*R*[*F*^2^ > 2σ(*F*^2^)] = 0.045	Weighting scheme based on measured s.u.'s *w* = 1/[σ^2^(*I*) + 0.0016*I*^2^]
*wR*(*F*^2^) = 0.109	(Δ/σ)_max_ = 0.013
*S* = 1.93	Δρ_max_ = 0.28 e Å^−^^3^
2225 reflections	Δρ_min_ = −0.21 e Å^−^^3^
189 parameters	Absolute structure: Flack (1983), 915 Friedel pairs
0 restraints	Flack parameter: 0.06 (2)
65 constraints	

### Special details

**Table:**

Refinement. The refinement was carried out against all reflections. The conventional *R*-factor is always based on *F*. The goodness of fit as well as the weighted *R*-factor are based on *F* and *F*^2^ for refinement carried out on *F* and *F*^2^, respectively. The threshold expression is used only for calculating *R*-factors *etc*. and it is not relevant to the choice of reflections for refinement.The program used for refinement, Jana2006, uses the weighting scheme based on the experimental expectations, see _refine_ls_weighting_details, that does not force *S* to be one. Therefore the values of *S* are usually larger than the ones from the *SHELX* program.

### Fractional atomic coordinates and isotropic or equivalent isotropic displacement parameters (Å^2^)

**Table:**

	*x*	*y*	*z*	*U*_iso_*/*U*_eq_	
Cl1	0.00790 (13)	0.16442 (1)	0.00937 (6)	0.0366 (3)	
O1	1.2823 (3)	0.6425 (3)	0.64185 (15)	0.0252 (7)	
O2	1.4726 (3)	0.8288 (3)	0.53638 (16)	0.0255 (7)	
O3	1.3717 (3)	0.8527 (3)	0.32476 (16)	0.0298 (8)	
N1	0.7360 (4)	0.5116 (4)	0.2085 (2)	0.0261 (10)	
C1	1.2081 (4)	0.6506 (4)	0.5342 (2)	0.0199 (9)	
C2	1.3141 (5)	0.7435 (4)	0.4792 (2)	0.0220 (10)	
C3	1.2528 (5)	0.7593 (4)	0.3685 (3)	0.0226 (11)	
C4	1.0855 (4)	0.6828 (4)	0.3111 (2)	0.0239 (10)	
C5	0.9789 (5)	0.5918 (4)	0.3668 (2)	0.0212 (10)	
C6	1.0392 (5)	0.5747 (4)	0.4767 (2)	0.0226 (11)	
C7	0.7985 (5)	0.5127 (4)	0.3104 (2)	0.0226 (10)	
C8	0.5578 (5)	0.4334 (4)	0.1638 (2)	0.0240 (10)	
C9	0.4039 (4)	0.4350 (4)	0.2092 (2)	0.0250 (11)	
C10	0.2330 (5)	0.3568 (4)	0.1612 (2)	0.0263 (11)	
C11	0.2161 (5)	0.2724 (4)	0.0662 (2)	0.0273 (11)	
C12	0.3676 (5)	0.2727 (4)	0.0172 (3)	0.0324 (12)	
C13	0.5356 (5)	0.3535 (5)	0.0654 (2)	0.0315 (12)	
C14	1.1767 (5)	0.5531 (5)	0.7017 (3)	0.0370 (13)	
C15	1.6479 (5)	0.7425 (5)	0.5441 (3)	0.0319 (12)	
C16	1.3098 (5)	0.8853 (5)	0.2132 (3)	0.0366 (14)	
H4	1.043968	0.692286	0.234845	0.0286*	
H6	0.964788	0.510597	0.51341	0.0271*	
H9	0.415976	0.491463	0.275474	0.03*	
H10	0.126966	0.360818	0.193233	0.0316*	
H12	0.354653	0.217079	−0.049398	0.0389*	
H13	0.638975	0.355215	0.031235	0.0378*	
H14a	1.230813	0.572264	0.776497	0.0444*	
H14b	1.044923	0.588152	0.682085	0.0444*	
H14c	1.182975	0.43867	0.686918	0.0444*	
H15a	1.753739	0.805308	0.585471	0.0382*	
H15b	1.643203	0.638974	0.578074	0.0382*	
H15c	1.664554	0.725127	0.473844	0.0382*	
H16a	1.401652	0.954863	0.192956	0.0439*	
H16b	1.299241	0.784387	0.174383	0.0439*	
H16c	1.186828	0.938464	0.197126	0.0439*	
H7	0.723679	0.459098	0.351803	0.0271*	

### Atomic displacement parameters (Å^2^)

**Table:**

	*U*^11^	*U*^22^	*U*^33^	*U*^12^	*U*^13^	*U*^23^
Cl1	0.0321 (4)	0.0363 (4)	0.0337 (4)	−0.0062 (4)	−0.0049 (3)	−0.0031 (4)
O1	0.0277 (12)	0.0274 (12)	0.0184 (10)	−0.0043 (10)	0.0021 (8)	−0.0007 (9)
O2	0.0210 (12)	0.0256 (12)	0.0276 (11)	−0.0031 (9)	0.0023 (9)	−0.0061 (9)
O3	0.0298 (13)	0.0353 (12)	0.0235 (11)	−0.0092 (11)	0.0054 (10)	0.0014 (10)
N1	0.0248 (16)	0.0292 (15)	0.0227 (15)	−0.0020 (12)	0.0032 (12)	−0.0012 (11)
C1	0.0216 (16)	0.0189 (14)	0.0184 (14)	0.0012 (14)	0.0039 (11)	−0.0004 (13)
C2	0.0190 (16)	0.0203 (15)	0.0239 (17)	−0.0012 (13)	0.0007 (13)	−0.0033 (12)
C3	0.0235 (18)	0.0192 (17)	0.0280 (17)	−0.0008 (13)	0.0115 (14)	0.0002 (12)
C4	0.0229 (17)	0.0235 (17)	0.0235 (15)	0.0008 (14)	0.0030 (12)	0.0005 (13)
C5	0.0182 (18)	0.0199 (15)	0.0251 (17)	0.0011 (12)	0.0046 (14)	−0.0007 (12)
C6	0.0239 (19)	0.0209 (17)	0.0227 (17)	0.0018 (13)	0.0057 (13)	0.0015 (12)
C7	0.0211 (17)	0.0200 (16)	0.0251 (17)	−0.0003 (13)	0.0033 (13)	−0.0005 (13)
C8	0.0263 (17)	0.0220 (16)	0.0206 (16)	−0.0015 (15)	0.0007 (13)	−0.0004 (13)
C9	0.0252 (18)	0.0281 (18)	0.0201 (16)	0.0027 (15)	0.0032 (13)	0.0004 (13)
C10	0.0254 (19)	0.0296 (18)	0.0219 (17)	0.0010 (14)	0.0024 (13)	0.0003 (14)
C11	0.034 (2)	0.0230 (16)	0.0191 (16)	0.0008 (14)	−0.0037 (14)	0.0007 (12)
C12	0.032 (2)	0.0353 (19)	0.0270 (18)	0.0025 (16)	0.0020 (15)	−0.0050 (14)
C13	0.032 (2)	0.040 (2)	0.0226 (17)	−0.0018 (16)	0.0080 (14)	−0.0030 (15)
C14	0.035 (2)	0.048 (2)	0.0262 (19)	−0.0060 (18)	0.0048 (15)	0.0083 (16)
C15	0.0185 (18)	0.0334 (18)	0.040 (2)	0.0021 (15)	0.0018 (15)	0.0018 (15)
C16	0.042 (2)	0.043 (2)	0.0260 (18)	−0.0086 (17)	0.0097 (16)	0.0074 (15)

### Geometric parameters (Å, °)

**Table:**

Cl1—C10	2.708 (3)	C7—H7	0.96
Cl1—C11	1.732 (3)	C8—C9	1.384 (5)
Cl1—C12	2.714 (4)	C8—C13	1.406 (5)
O1—C1	1.362 (3)	C9—C10	1.383 (4)
O1—C14	1.423 (5)	C9—H9	0.96
O2—C2	1.379 (4)	C10—C11	1.390 (5)
O2—C15	1.427 (4)	C10—H10	0.96
O3—C3	1.375 (4)	C11—C12	1.398 (6)
O3—C16	1.424 (4)	C12—C13	1.378 (5)
N1—C7	1.282 (4)	C12—H12	0.96
N1—C8	1.417 (4)	C13—H13	0.96
C1—C2	1.397 (5)	C14—H14a	0.96
C1—C6	1.397 (4)	C14—H14b	0.96
C2—C3	1.395 (4)	C14—H14c	0.96
C3—C4	1.390 (4)	C15—H15a	0.96
C4—C5	1.398 (5)	C15—H15b	0.96
C4—H4	0.96	C15—H15c	0.96
C5—C6	1.385 (4)	C16—H16a	0.96
C5—C7	1.466 (4)	C16—H16b	0.96
C6—H6	0.96	C16—H16c	0.96
			
C10—Cl1—C12	52.95 (11)	C8—C9—H9	119.2976
C1—O1—C14	117.5 (2)	C10—C9—H9	119.2983
C2—O2—C15	112.4 (3)	C9—C10—C11	119.5 (3)
C3—O3—C16	117.3 (2)	C9—C10—H10	120.2657
C7—N1—C8	117.6 (3)	C11—C10—H10	120.265
O1—C1—C2	115.4 (3)	C10—C11—C12	120.2 (3)
O1—C1—C6	125.5 (3)	C11—C12—C13	119.5 (3)
C2—C1—C6	119.0 (3)	C11—C12—H12	120.2721
O2—C2—C1	119.1 (3)	C13—C12—H12	120.2719
O2—C2—C3	120.3 (3)	C8—C13—C12	120.9 (4)
C1—C2—C3	120.5 (3)	C8—C13—H13	119.5347
O3—C3—C2	114.3 (3)	C12—C13—H13	119.5353
O3—C3—C4	125.1 (3)	O1—C14—H14a	109.4713
C2—C3—C4	120.5 (3)	O1—C14—H14b	109.4711
C3—C4—C5	118.6 (3)	O1—C14—H14c	109.4713
C3—C4—H4	120.6757	H14a—C14—H14b	109.4712
C5—C4—H4	120.6756	H14a—C14—H14c	109.4714
C4—C5—C6	121.2 (3)	H14b—C14—H14c	109.471
C4—C5—C7	120.8 (3)	O2—C15—H15a	109.4714
C6—C5—C7	118.0 (3)	O2—C15—H15b	109.4711
C1—C6—C5	120.1 (3)	O2—C15—H15c	109.4715
C1—C6—H6	119.9695	H15a—C15—H15b	109.4705
C5—C6—H6	119.9698	H15a—C15—H15c	109.4714
N1—C7—C5	123.1 (3)	H15b—C15—H15c	109.4714
N1—C7—H7	118.4336	O3—C16—H16a	109.4709
C5—C7—H7	118.4335	O3—C16—H16b	109.4714
N1—C8—C9	124.2 (3)	O3—C16—H16c	109.4711
N1—C8—C13	117.3 (3)	H16a—C16—H16b	109.4712
C9—C8—C13	118.4 (3)	H16a—C16—H16c	109.4712
C8—C9—C10	121.4 (3)	H16b—C16—H16c	109.4715

### Hydrogen-bond geometry (Å, °)

**Table:**

*D*—H···*A*	*D*—H	H···*A*	*D*···*A*	*D*—H···*A*
C7—H7···O1^i^	0.96	2.59	3.177 (4)	119
C7—H7···O2^i^	0.96	2.51	3.471 (4)	178
C12—H12···N1^ii^	0.96	2.61	3.545 (5)	164

Symmetry codes: (i) −*x*+2, *y*−1/2, −*z*+1; (ii) −*x*+1, *y*−1/2, −*z*.
